# A new sister journal to the *Journal of Human Genetics*—for the interest and benefit of the global community of human genome researchers

**DOI:** 10.1038/hgv.2014.3

**Published:** 2014-07-24

**Authors:** Katsushi Tokunaga

**Affiliations:** 1 Department of Human Genetics, Graduate School of Medicine, The University of Tokyo, Bunkyo-ku, Tokyo, Japan

I am very pleased to see our new journal *Human Genome Variation* (*HGV*) has been launched as the sister journal to the *Journal of Human Genetics* (*JHG*), an official journal of the Japan Society of Human Genetics, with the aim of creating a new open publishing platform for quality, fully peer-reviewed papers on human genome research, and the collection of information on the newly reported gene variants.

*HGV* has a similar scope to its sister journal *JHG* but has a greater focus on studies and discoveries of genome variation or human disease-causable mutation. The journal was born in response to the strong demand by the community for a place to publish important discoveries, observations and analysis about research on the human genome in the era of personal and clinical sequencing. In recent years we see most journals are selecting and publishing papers of major perceived impact (or perceived likeliness of getting cited). Those papers reporting a smaller number of gene variants, which are novel and of value, tend not to be published. The team that I led decided we needed to find a way to publish those papers—moreover, to publish those papers on a well-established and reputable platform and make them widely available to all. *HGV* is the result of our thinking, discussion with the community and collaboration with our valued colleagues and partners.

In developing ideas for the new journal we have created a short article type called the ‘Data Report’. Data Reports will be a core part of the journal, collecting and presenting peer-reviewed information of newly found or newly studied genetic variants. Key information from published Data Reports will be added to the ‘*HGV* Database’, which is being launched as part of *HGV* journal (in partnership with Figshare). The *HGV* Database will become a rich trove of linked, organized data and readers will be able to search, filter and locate genome variation. *HGV* and the *HGV* Database aim to build and deliver a set of information on newly discovered human disease-causative gene variations from different regions of the world whose value grows over time. We believe that formalizing an explicit link between the peer-reviewed article and analysis, and the data underlying the article will be a useful, valuable and vital source of information for the global community of genome researchers, clinical geneticists and genetic counselors.

*Human Genome Variation* will also publish peer-reviewed original articles (known as Articles) and Review Articles. Selected accepted articles will be accompanied by an Editorial Summary, which is a professionally written, short synopsis of an article. Editorial Summaries are written with a broader audience in mind, so providing increased accessibility, increased readership and increased awareness of each article. Authors are able to re-use and e-mail Editorial Summaries to highlight the value of their work.

We are grateful to the Japan Society of Promotion of Science (JSPS) for their generous support to help us launch *Human Genome Variation*, which is designed to have functional and explicit links between the written article, the data and the accessible summary. We hope that *Human Genome Variation* will contribute to the advancement of human genome and related studies.

On behalf of the entire editorial team, I welcome you to *Human Genome Variation* and look forward to presenting the latest research to facilitate discussion and collaboration that will continue the advancements in the field of human genome science. We encourage you to submit Articles and Data Reports, assist with peer review when requested and of course read and utilize the resources we are developing—and so become an integral part of the success of *Human Genome Variation*.

